# Accelerometer-measured and self-reported physical activity in relation to extraversion and neuroticism: a cross-sectional analysis of two studies

**DOI:** 10.1186/s12877-020-01669-7

**Published:** 2020-07-29

**Authors:** Tiia Kekäläinen, Eija K. Laakkonen, Antonio Terracciano, Tiina Savikangas, Matti Hyvärinen, Tuija H. Tammelin, Timo Rantalainen, Timo Törmäkangas, Urho M. Kujala, Markku Alen, Vuokko Kovanen, Sarianna Sipilä, Katja Kokko

**Affiliations:** 1 Gerontology Research Center and Faculty of Sport and Health Sciences, University of Jyväskylä, P.O. Box 35, 40014 Jyväskylä, Finland; 2 Department of Geriatrics, College of Medicine, Florida State University, Tallahassee, FL USA; 3 LIKES Research Centre for Physical Activity and Health, Jyväskylä, Finland; 4 Faculty of Sport and Health Sciences, University of Jyväskylä, Jyväskylä, Finland; 5 Oulu University Hospital, Oulu, Finland

**Keywords:** Personality, Traits, Accelerometer, Exercise, Leisure time

## Abstract

**Background:**

Personality reflects relatively stable and pervasive tendencies in feeling, thinking and behaving. While previous studies have found higher extraversion and lower neuroticism to be linked to higher self-reported physical activity levels, larger studies using accelerometer-measured physical activity are lacking. This study investigated the cross-sectional associations of extraversion and neuroticism with both accelerometer-measured and self-reported physical activity and the role of these personality traits in possible discrepancies between these two measures of physical activity among Finnish adults.

**Methods:**

Two community-dwelling samples were used in this study: a) 47–55-yr-old women (*n* = 1098) and b) 70–85-yr-old women and men (*n* = 314). In both samples, extraversion and neuroticism were assessed by the 19-item short form of the Eysenck Personality Inventory. Physical activity was assessed with hip-worn tri-axial accelerometers and self-reported questions. Regression analyses were adjusted by age, BMI and education.

**Results:**

In the middle-aged women, neuroticism was negatively associated with accelerometer-measured leisure time moderate-to-vigorous physical activity (β = −.07, *p* = .036) and with self-reported physical activity (β = −.08, *p* = .021), while extraversion was positively associated with self-reported physical activity (β = .10, *p* = .005). No associations of extraversion or neuroticism with physical activity were found in the older men and women. Older adults who scored high in neuroticism reported less physical activity than what was measured by accelerometers (β = −.12, *p* = .039). Extraversion was not associated with discrepancy between self-reported and accelerometer-measured leisure time physical activity in either sample.

**Conclusions:**

Neuroticism was associated with lower leisure-time physical activity levels and extraversion with higher self-reported physical activity among middle-aged women. Neuroticism and extraversion were unrelated to physical activity among older adults, but older adults with high neuroticism seemed to underreport their physical activity level. The role of personality in the discrepancy between self-reported and device-based physical activity warrants further research.

## Background

A notable proportion of adults are globally regarded as physically inactive [1]. Population-based studies have also shown that physical activity levels are lower in older age groups (60+) [2–4]. This is concerning, as regular participation in physical activity helps to reduce many negative age-related changes in the human body, such as the decline in muscle strength, bone density and cardiovascular fitness [5]. In women, menopause is associated with worsening of physical performance [6, 7] and decline in physical activity levels [8]. Regular physical activity could help to prevent negative menopausal-related changes, such as muscle strength and power loss and increased visceral adiposity [7, 8].

To promote physically active lifestyle, it is crucial to identify the factors associated with physical activity and inactivity in different stages of life. Among the potential psychological factors, pervasive individual differences in feeling, thinking and behaving, called personality [9], may underlie an individual’s willingness to be physically active and help identify risk groups for inactivity.

Individual differences in personality traits are known to be relatively stable throughout the adult lifespan [10]. However, some longitudinal studies indicate that extraversion declines with age, whereas neuroticism typically declines from early to middle-adulthood and may slightly increase thereafter [11]. Personality traits may shape a multitude of health behaviors, including physical activity [12–14]. Previous meta-analyses have shown that adults scoring high on extraversion (i.e., a tendency to be outgoing, sociable, active, and assertive) and low on neuroticism (i.e., the tendency to experience negative emotions, such as anxiety, tension and self-pity) report higher levels of physical activity [12–14].

Most previous studies have used self-reported measures of physical activity. Correlations between self-reported and device-based physical activity measures have been low to moderate [15, 16], indicating that they measure partly different aspects of physical activity. Thus, including device-based physical activity measures may yield new knowledge in research on personality and physical activity. The strengths of device-based physical activity measures, such as accelerometers, include minute-by-minute monitoring, also of light intensity physical activity that is often underreported in questionnaires [17]. This is important, especially among older adults whose daily activity is mainly light intensity [17]. Accelerometer studies have not reported consistent associations with personality traits [18–22]. This may be due to relatively small sample sizes and the use of convenience samples of active older adults (*n* = 69) [20], obese middle-aged men and women (*n* = 235) [18], college women (*n* = 294) [19] and young men and women (*n* = 64) [22].

However, hip-worn accelerometers have their limitations. For example, hip-worn devices imperfectly capture cycling or upper-body exercises [17, 23]. In addition, the thresholds for different intensities of physical activity (light, moderate, vigorous) do not take individuals’ fitness levels into account [17, 24]: accelerometer-measured light activity, for example, may be experienced as very strenuous by low-fit individuals. It is, therefore, important to measure physical activity in multiple ways. Moreover, although accelerometers and questionnaires do not measure exactly the same aspect of physical activity, personality may play a role in how people estimate and report their physical activity. Individuals high in extraversion may over-report and individuals high in neuroticism under-report their physical activity levels. It has been found, for example, that individuals who scored high in neuroticism were more likely to over-estimate their weight, whereas those higher in extraversion typically assessed themselves as leaner and taller than they actually are [25]. To our knowledge, the associations of both accelerometer-measured and self-reported physical activity with personality traits have simultaneously been investigated in only one previous study, which found that, among college women, extraversion was associated only with higher self-reported physical activity whereas neuroticism was associated only with lower moderate-to-vigorous accelerometer-measured physical activity [19].

This study aimed to contribute to this growing area of research by exploring a) the cross-sectional associations of extraversion and neuroticism with both accelerometer-measured and self-reported physical activity and b) the role of extraversion and neuroticism in discrepancies between accelerometer-measured and self-reported physical activity. This study is based on two relatively large samples of Finnish adults, which represent two groups at risk for physical inactivity: menopausal women and sedentary or at most moderately physically active older adults.

## Methods

### Participants

**Fig. 1:**
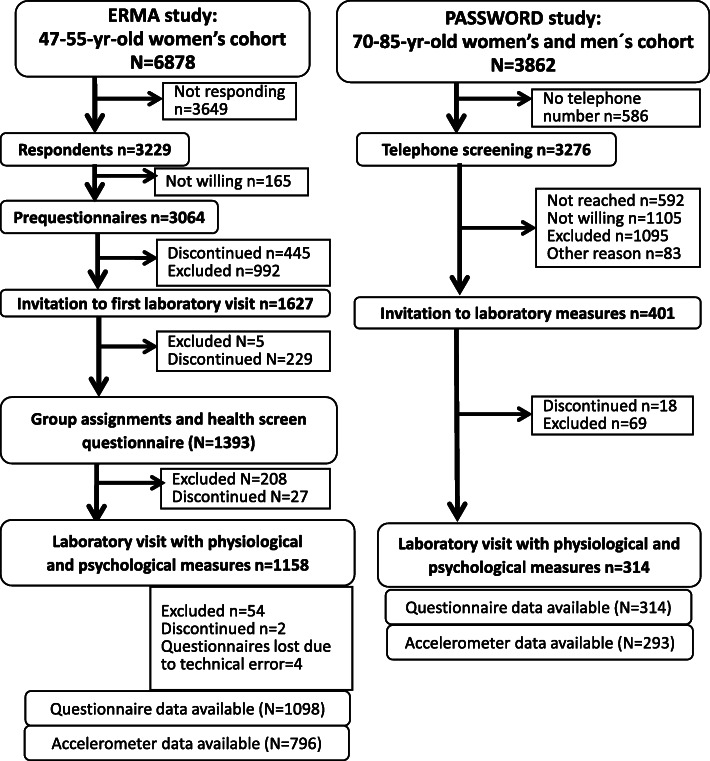
Flow chart of the studies

Participants were drawn from the baseline samples of two research projects: “Estrogenic Regulation of Muscle Apoptosis” (the ERMA study) [26] and “Promoting safe walking among older people: the effects of a physical and cognitive training intervention vs. physical training alone on mobility and falls among older community-dwelling men and women” (the PASSWORD study) [27]. Both the ERMA and the PASSWORD studies were approved by the ethics committee of the Central Finland Health Care District (Dnro 8 U/2014 and 11/2016, respectively) and all participants provided their written informed consent prior to participation. In both studies, participants were randomly selected from the Finnish National Registry. The study designs and recruitment of participants are described in previously published papers [26–28]. The flow chart of the studies is shown in Fig. 1. More information about representativeness of the samples can be found from Additional file 1.

*The ERMA study* [26]: Participants were 47- to 55-year-old women living in the city of Jyväskylä or neighboring municipalities in Finland. Exclusion criteria were self-reported body mass index (BMI) > 35, being currently pregnant or lactating, conditions or medications affecting ovarian function, and chronic diseases or medications affecting muscle function. The initial sample comprised 6878 participants. Of these, 47% (*n* = 3229) responded to the postal invitation and 1393 women provided blood samples to allow screening of their menopausal status. The baseline questionnaire was available from 1098 participants. The recruitment occurred from November, 2014 to July, 2018 and the baseline data were collected between February, 2015 and November, 2018.

*The PASSWORD study* (ISRCTN52388040) [27]: Participants were community-dwelling 70- to 85-year-old men and women living in the city of Jyväskylä, Finland. Inclusion criteria were sedentary or at most moderately active (walking < 150 min/wk., no regular resistance training), able to walk 500 m without assistance and a score of ≥24 points in a Mini Mental State Examination [23]. Exclusion criteria included a severe chronic condition, medication, or other factors that could affect study participation, excessive use of alcohol, difficulties in communication due to severe hearing or vision problems, and another family member already participating in the study. The initial sample comprised 3862 participants [28]. Of these, 69% (*n* = 2684) were reached by telephone for the screening interview. Of this number, 1178 were excluded for health-related reasons, being excessively active or for some other reason, and 1105 declined to participate. The remaining 401 participants were invited to attend the laboratory measurements. Clinical exclusion criteria were assessed, and health status confirmed by a nurse and, if necessary, a physician and a clinical psychologist before the baseline assessments. The final sample was 314. The recruitment occurred from January, 2017 to March, 2018, and the baseline data were collected between February, 2017 and March, 2018.

### Measures

**Extraversion and neuroticism** were assessed in both studies with the 19-item short form of the Eysenck Personality Inventory developed by Floderus [29, 30]. Nine items (e.g. *Are you lively and talkative?*) assessed extraversion and 10 items (e.g. *Do you often feel apathetic and tired without any special reason?*) assessed neuroticism using a dichotomous response scale (0 = no and 1 = yes). Sum scores were calculated for both extraversion and neuroticism. Because a missing value may result in sum scores that are too low, the probability of a positive response was calculated for participants who had missing values (less than 5% of participants) and was then used to calculate the imputed sum scores (e.g. if one value on neuroticism was missing, that value was imputed with the option 1 = yes for participants who scored five or more from the remaining nine items on the neuroticism scale). Cronbach’s alphas were 0.79 for extraversion and 0.65 for neuroticism in the ERMA study and 0.77 for extraversion and 0.72 for neuroticism in the PASSWORD study. Deleting any of the items would not have increased the alpha values in either sample.

**Self-reported physical activity** was assessed by two different sets of questions focusing mainly on leisure time physical activity in both studies. The first was a single question on the participant’s current physical activity level with seven response categories: 1 = *I do not move more than is necessary in my daily routines/chores,* 2 = *I go for casual walks and engage in light outdoor recreation 1–2 times a week*, 3 = *I go for casual walks and engage in light outdoor recreation several times a week*, 4 = *I engage 1–2 times a week in brisk physical activity (*e.g. *yard work, walking, cycling) to the point of perspiring and some degree of breathlessness*, 5 = *Several times a week (3–5) I engage in brisk physical activity (*e.g. *yard work, walking, cycling) to the point of perspiring and some degree of breathlessness*, 6 = *I do keep-fit exercises several times a week in a way that causes rather strong shortness of breath and sweating during the activity*, and 7 = *I participate in competitive sports and maintain my fitness through regular training* [31]. Participants were asked to select the description that best corresponds to their current level of physical activity. The variable was recoded into three categories: *1 = low* (categories 1 and 2), *2 = medium* (categories 3 and 4) and *3 = high* (categories 5 to 7) [32]. The second set of questions comprised three items on the frequency (Currently, how many times per month do you engage in physical activity during your leisure-time?), mean intensity (Is your leisure-time physical activity about as tiring on average as...) and mean duration (How long does your average session of leisure-time physical activity last?) of leisure time exercise [33]. The ERMA participants also answered an item on the time they spent walking, cycling or running during their daily journey to work. Responses were recorded as MET minutes.

**Accelerometer-measured physical activity** was measured with different tri-axial accelerometers in the two samples [28, 32]. In both samples, participants were advised to wear the monitors on the right side of their pelvis (just below the iliac crest at the height of the spina iliaca anterior superior) during waking hours, except for water-based activities, for seven consecutive days. Participants were instructed to keep a diary of their accelerometer wear time and periods when the accelerometer was removed for over 30 min. For accelerometer data to be acceptable, a commonly used threshold of wear time of at least 10 h per day on at least 3 days was required [34].

The ERMA study: GT3X+ and wGT3X+ ActiGraph accelerometers (Pensacola, FL) were used. Raw acceleration data were collected at 60 Hz, filtered and converted into 60-s epoch counts. Tri-axial vector magnitude cut-points of 450 cpm, 2690 cpm, and 6166 cpm were used to separate sedentary time and light, moderate and vigorous intensity physical activity [32, 35, 36]. Moderate and vigorous intensity physical activity were combined for further analyses (MVPA). Working time and leisure time were distinguished by utilizing diary data. Acceptable accelerometer data were available for 796 women.

The PASSWORD study: UKK RM42 (UKK, Tampere, Finland) accelerometers were used. Raw acceleration data were collected at a 100 Hz sampling rate with 13-bit A/D conversion of the ±16 g range and analyzed with a custom-written MATLAB (version R2016b, The MathWorks Inc., Natick MA, USA) script for mean amplitude deviation (MAD) in non-overlapping 5 s epochs [37]. Twelve consecutive 5 s epochs were averaged to produce minute-by-minute MADs. Previously defined and validated cut-points of 16.7 mg (milli acceleration caused by gravity), 91 mg and 414 mg were used to categorize the minutes into separate sedentary time and light, moderate and vigorous intensity physical activity [37, 38]. The number of minutes per day spent in each category were calculated and the time spent in moderate and vigorous intensity activities combined and used as the outcome (MVPA). In total, 293 participants had acceptable accelerometer data and were included in the present analyses.

Because different methods (ActiGraph counts and MAD) were used to analyze the raw accelerometer data in the studies, the absolute levels of light and MVPA are not directly comparable between the two samples [39]. Therefore, the subsequent analyses were conducted independently for each sample.

**Background variables** included sex, age, BMI and education. Sex and date of birth were drawn from the Finnish Population Registry. BMI was calculated from height and weight measured by the study nurse. Highest level of education was self-reported in the questionnaires and categorized into 1 = lower (less than university degree) and 2 = higher (university degree).

Additionally, information on menopausal status and employment status from the ERMA study and on chronic diseases and walking speed from both the ERMA and the PASSWORD studies were used in the sensitivity analyses. In the ERMA study, women were categorized into a pre-, peri- or post-menopausal group based on bleeding diaries and hormonal levels [26, 36]. Employment status was dichotomized into the categories employed (paid or self-employed) and other (student, unemployed, working occasionally, retired, taking care of the home) [36]. Chronic diseases diagnosed by physicians were self-reported. In the PASSWORD study, data collected from the National Health Service integrated patient information system and in a clinical examination were used together with the self-reported data. Chronic diseases were categorised into metabolic, cardiovascular, pulmonary, musculoskeletal, neurological and mental disorders. The number of disease categories was calculated and coded for the analyses as 0, 1, 2 and 3 or more disease categories. Maximal walking speed over 10 m was measured in both the ERMA and the PASSWORD studies and the best performance of two trials was used as a result [7, 27].

### Statistical analyses

All statistical analyses were carried out using SPSS, version 25 (IBM Corp., Armonk, NY). Statistical significance was set at *p* < 0.05. The descriptive statistics are presented with frequencies or means and standard deviations.

Associations between personality traits and physical activity were analyzed in both samples using similar regression analyses. Fulfilment of the linearity assumption was checked by graphical inspection before the linear regression analysis. Pairwise deletion was used to handle missing values. Separate linear regression analyses were conducted for the outcomes of accelerometer-measured light physical activity and MVPA and for self-reported MET-minutes. To allow full comparison between the different studies representing different age groups, the accelerometer-measured physical activity data obtained in the ERMA study was separately analyzed as whole-day time, which also included occupational physical activity, and as leisure time physical activity excluding occupational physical activity. Because of skewed distributions in the physical activity variables, square root transformation of accelerometer-assessed MVPA variables and self-reported MET-minutes in the ERMA study and cube root transformation of self-reported MET-minutes in the PASSWORD study were used in regression analyses. Ordinal regression was used for the self-reported physical activity categories after the assumption of proportional odds was confirmed by non-significant tests of parallel lines. Because extraversion and neuroticism correlated moderately with each other (r = −.37, *p* < .001 in both samples), they were analyzed in separate models. To gain more insight into their joint association with physical activity, the models with extraversion and neuroticism in the same model are presented in the supporting information (Table S3, Additional file 2). Additionally, the Wald test was used to analyze the joint significance of extraversion and neuroticism with the physical activity variables (Table S4, Additional file 2).

All regression analyses were adjusted for age, BMI and education, variables which are commonly associated with physical activity and personality traits. All analyses for accelerometer-measured outcomes were adjusted for accelerometer wear time and the model for light physical activity for MVPA and vice versa. The analyses with the PASSWORD sample were also adjusted for sex. To see whether sex plays any role in the studied associations, the moderator effect of sex was analyzed in the PASSWORD sample by adding sex*trait interaction terms into the models (Table S6, Additional file 2). Additional sensitivity analyses were conducted to adjust the analyses for the ERMA sample for menopausal status, chronic diseases and walking speed and for the PASSWORD sample for chronic diseases and walking speed. The sensitivity analyses for the ERMA sample for leisure time physical activity were also adjusted for employment status (Table S5, Additional file 2). Other lifestyle factors were excluded from consideration as covariates due to the potential role of personality traits as determinants of lifestyle variables.

Because self-reported MET minutes were calculated based on questions about leisure time physical activity, the discrepancy comparison was made between self-reported MET minutes and accelerometer-measured leisure time MVPA. Since these measures are not directly comparable, the discrepancy between their scores was calculated to indicate whether individuals who reported the most leisure time physical activity also showed the most accelerometer-measured MVPA. For participants for whom both self-reported MET minutes and accelerometer-measured leisure time MVPA were available (*n* = 789 in the ERMA sample and *n* = 293 in the PASSWORD sample), the difference between the standardized variables was calculated. Standardized accelerometer-measured leisure time MVPA was subtracted from standardised self-reported MET minutes. A positive discrepancy score indicates that compared to all the other participants, an individual had reported more physical activity than recorded by the accelerometer. The associations of personality traits with the discrepancy scores were analysed with regression analyses similar to those described above.

## Results

### Descriptive statistics

The descriptive statistics for the two samples are presented in Table 1. In the ERMA study, the participants who did not provide acceptable accelerometer data or BMI data were younger and scored higher in neuroticism compared to those from whom acceptable accelerometer data or BMI data were available (Table S2, Additional File 1). In the PASSWORD sample, women were overrepresented in the group which did not provide acceptable accelerometer data (Table S2, Additional File 1). The correlations between self-reported MET-minutes and accelerometer-measured leisure time MVPA were *r* = .33, *p* < .001 and *r* = .27, *p* < .001, respectively, in the ERMA and PASSWORD samples.

**Table 1:** Descriptive statistics for the ERMA and the PASSWORD participants

	ERMA study (*n* = 1098)			PASSWORD study (*n* = 314)	
	Mean ± SD		n	Mean ± SD	n
Sex (female), n (%)	1098 (100)		1098	188 (59.9)	314
Age, yrs	50.9 ± 2.1		1098	74.5 ± 3.8	314
BMI	25.5 ± 3.7		927	27.9 ± 4.7	314
Education (higher), n (%)	455 (41.4)		1098	66 (21.0)	314
Extraversion	5.4 ± 2.7		1095	4.5 ± 2.7	314
Neuroticism	2.9 ± 2.2		1095	3.2 ± 2.3	314
Self-reported physical activity categories			1098		314
Low n (%)	120 (10.9)			126 (40.1)	
Medium n (%)	297 (27.0)			148 (47.1)	
High n (%)	681 (62.0)			40 (12.7)	
Self-reported physical activity, MET-min/day	271.4 ± 234.8		1091	79.4 ± 107.9	313
Accelerometer-measured physical activity	Leisure time	Whole-day	796	Whole-day	293
Light physical activity min/day	221.7 ± 68.2	295.2 ± 76.7		210.3 ± 66.3	
MVPA min/day	43.0 ± 24.0	49.7 ± 25.9		32.5 ± 20.1	
Wear time h/day	11.3 ± 2.4	15.2 ± 1.0		14.1 ± 1.3	

### Associations between personality traits and physical activity

The results of the regression analyses for both samples are presented in Table 2. Extraversion and neuroticism were not associated with accelerometer-measured whole-day or leisure-time light physical activity in either sample. Among middle-aged women, a negative association was observed between neuroticism and accelerometer-measured leisure-time MVPA, but not for whole-day MVPA. Extraversion was not associated with accelerometer-measured whole-day or leisure-time MVPA in either sample. Extraversion and neuroticism did not have a statistically significantly joint association with any accelerometer-measured physical activity outcome (Table S4, Additional file 2).

**Table 2:** Associations of personality traits with physical activity in middle-aged women (ERMA) and older adults (PASSWORD)

	Accelerometer-measured physical activity								Self-reported physical activity			
	Leisure time				Whole-day				Leisure time			
	Light PA		MVPA		Light PA		MVPA		MET		PA categories^a^	
	β	p	β	p	Β	p	β	p	β	p	OR	95% CI
**Unadjusted models**												
ERMA study												
Extraversion	.01	.860	.05	.131	.01	.881	.06	.099	.09	.016	1.04	1.00–1.09
Neuroticism	.02	.533	−.08	.030	.03	.429	−.06	.086	−.10	.006	0.91	.86–.96
PASSWORD study												
Extraversion					.05	.441	−.03	.629	−.06	.289	.95	.88–1.03
Neuroticism				−.01		.833	.04	.461	−.05	.401	.98	.89–1.07
**Adjusted models**^**b**^												
ERMA study												
Extraversion	.00	.902	.06	.076	.01	.754	.07	.055	.10	.005	1.06	1.01–1.11
Neuroticism	.02	.443	−.07	.036	.03	.350	−.05	.163	−.08	.021	0.92	.86–.98
PASSWORD study												
Extraversion					.07	.185	−.01	.814	−.04	.496	.96	.88–1.04
Neuroticism					.01	.927	.09	.108	−.04	.533	.99	.90–1.09

Extraversion and neuroticism were tested in separate regression models. Standardized Beta-coefficients (β) and *p*-values are presented for linear regression analyses and odds ratios (OR) with 95% confidence interval (CI) for ordinal regression models. *PA* Physical activity, *MVPA* Moderate-to-vigorous physical activity

^a^PA categories were low, medium and high (reference group)

^b^Models adjusted for age, BMI and education. Models for accelerometer-measured outcomes also adjusted for accelerometer wear time. The model for light PA adjusted for MVPA and the model for MVPA adjusted for light PA. Models for the PASSWORD data were also adjusted for sex

Among the middle-aged women, both extraversion and neuroticism were associated with self-reported physical activity outcomes: higher scores on extraversion and lower scores on neuroticism were associated with higher self-reported physical activity (Table 2). Similar associations were found for self-reported leisure time physical activity (MET-minutes) and the physical activity categories. The joint associations of extraversion and neuroticism with self-reported physical activity outcomes were also statistically significant (Table S4, Additional file 2). No associations between personality traits and self-reported physical activity were found among the older men and women.

### Sensitivity analyses

In the sensitivity analyses, the models for the ERMA study were additionally adjusted by menopausal status, chronic diseases and walking speed and the analyses for leisure time by employment status or adjusting the models for the PASSWORD study by chronic diseases and walking speed (Table S5, Additional file 2). These additional adjustments slightly attenuated the associations between neuroticism and physical activity: the associations of neuroticism with accelerometer-measured leisure time MVPA (β = −.06, *p* = .081), self-reported MET minutes (β = −.07, *p* = .053), and with the self-reported physical activity categories (OR 0.94 95% CI 0.88–1.01) were no longer statistically significant. The positive association between extraversion and both self-reported physical activity outcomes remained statistically significant. The interaction effect of sex was tested in the PASSWORD sample. None of the interaction terms were statistically significant (Table S6, Additional file 2), indicating that the associations were not significantly different between women and men.

### Associations of personality traits with the discrepancy between the physical activity measures

Extraversion was not associated with the discrepancy between scores in either sample (Table 3). Neuroticism was not associated with the discrepancy between scores in the middle-aged women; however, older adults scoring high in neuroticism reported less physical activity than that assessed by their accelerometers. This association was statistically significant after adjustment for age, sex, education and BMI.

**Table 3:** Associations of personality traits with discrepancy between physical activity measures

	Unadjusted models		Adjusted models^a^		Adjusted models^b^	
	β	p	β	p	β	p
ERMA study						
Extraversion	.04	.298	.04	.251	.04	.262
Neuroticism	−.02	.668	−.01	.786	−.01	.845
PASSWORD study						
Extraversion	.01	.859	−.00	.939	.00	.964
Neuroticism	−.08	.171	−.12	.039	−.12	.038

The dependent variable was the difference between standardized self-reported MET minutes and accelerometer-measured leisure time MVPA (mean ± SD: −.03 ± 1.13 in the ERMA study and − .01 ± 1.21 in the PASSWORD study). Extraversion and neuroticism were tested in separate regression models

^a^Models adjusted for age, BMI and education and for the PASSWORD data also for sex

^b^Models adjusted also for accelerometer wear time, chronic diseases and walking time and for the ERMA data also for menopausal status

The PASSWORD sample showed one outlier with a high discrepancy score (11.67, second highest was 3.27). The results remained similar when the outlier was removed from the analysis. The sensitivity analyses were performed with additional confounders (Table 3). Models were adjusted by accelerometer wear time, chronic diseases and walking speed, and also by menopausal status for the ERMA participants. These additional adjustments had no effect on the associations between personality traits and the discrepancy between scores.

## Discussion

This study investigated whether extraversion and neuroticism are associated with accelerometer-measured and self-reported physical activity among two samples of Finnish adults: a sample of middle-aged women and a sample of older women and men. Among the middle-aged women, those who scored higher in extraversion and lower in neuroticism, reported higher levels of physical activity. Among these women, lower scores in neuroticism also had a weak negative association with accelerometer-measured leisure time MVPA. Neither of these associations were observed among the older adults. Among older adults, high neuroticism was associated with underreporting physical activity compared to accelerometer-assessed physical activity.

In line with the literature [12–14], extraversion had a positive association with self-reported physical activity among middle-aged women. Furthermore, as in previous studies among college women [19], obese middle-aged adults [18] and older adults [21], extraversion was not associated with accelerometer-measured physical activity. It is possible that people high in extraversion tend to give socially desirable responses to self-reports [19]. However, extraversion did not explain the discrepancy between self-reported and accelerometer-measured leisure time physical activity and a showed a tendency towards an association with accelerometer-measured leisure time MVPA (*p* = .076). However, extraversion seems to be related to leisure time exercise alone and not to light-intensity daily activities.

In line with previous studies [12–14], in the sample of middle-aged women, neuroticism was negatively associated with self-reported physical activity. As in the study among college women [19], this negative association was also seen with leisure-time accelerometer-measured MVPA. Although the association with accelerometer-measured MVPA was weak and would become statistically non-significant if *p*-values were corrected with the number of tests, the negative association of neuroticism in physical activity seems to be consistent across both self-reported and accelerometer-measured leisure time physical activity. Based on the sensitivity analysis (the association was attenuated from β = −.07 to β = −.06), this association seems to be at least partially explained by health status. Additionally, the association may have been underestimated, because women who scored high in neuroticism were less likely to provide acceptable accelerometer data. People who score high in neuroticism may have a tendency to avoid the intensive stimulation offered by physical activity [14]. The negative feelings that are often experienced by individuals who score higher on neuroticism, such as anxiety and depression [9], may also decrease the willingness to be physically active. However, physical activity interventions may help to reduce symptoms of anxiety and depression [40], and hence people with high neuroticism could potentially benefit from physical activity interventions. Further research should address ways of promoting physical activity among people who score high on neuroticism. For example, good barrier self-efficacy may help such individuals to overcome the negative effects of high neuroticism on physical activity [41].

Interestingly in this study, the negative association of neuroticism was only found with leisure time MVPA, and not with whole-day MVPA or whole-day or leisure time light physical activity. People high in neuroticism typically have lower occupational status [42] and may therefore be more physically active during their working hours. Moreover, personality may play a more important role in leisure time physical activity, as people are likely to have more control over their physical activity during their leisure time than work time. Given that leisure time and occupational physical activity may have different links with health [43] and that MVPA is more beneficial for health than lighter intensity activities [44], it would be important to pay more attention to neuroticism as a predictor of leisure time MVPA. However, due to the observational nature of this study, the causal relationship between neuroticism and leisure-time MVPA remains unclear and warrants further research.

The associations found among middle-aged women were not observed in the sample of older adults. This is surprising, since previous studies with large samples and meta-analyses suggest robust associations between personality traits and self-reported physical activity without a moderator effect of sex or age [13, 14]. However, similar finding was also reported by Čukić and colleagues [21], who found no associations between personality traits and accelerometer-measured step counts among older adults. The physical activity levels of the older adults in the PASSWORD sample showed a rather narrow range and low variance, as being sedentary or at most moderately active were among the inclusion criteria [27]. The small proportion participating in regular physical activities and sports is likely to explain the absence of any associations between personality traits and physical activity in this sample. Terracciano and colleagues [45], who studied a sample of US older adults, found that while personality traits had essentially no associations with resting metabolic rate and energy expenditure at normal walking pace, lower neuroticism and higher extraversion were associated with walking significantly faster and higher energy expenditure at peak walking pace. This pattern suggests that personality traits are less predictive of activity level in samples limited to individuals or activities with low energy expenditure.

To our knowledge, this was the first study to investigate the role of personality traits in discrepancies in the two different physical activity outcomes. A weak negative association between neuroticism and a discrepancy between scores was found among older adults. In line with previous results showing that individuals who score high in neuroticism are more likely to over-estimate their weight [25, 46], our finding suggests that such individuals may also tend to underestimate their level of physical activity. Neuroticism describes a predisposition to experience negative feelings, and hence this result may support this negative way of looking at life. This would be a fruitful area for future studies using more detailed personality questionnaires and more comparable physical activity measures.

Our discrepancy analysis was limited by the use of physical activity measures that are not directly comparable. The self-report items asked about mean monthly activity, whereas accelerometers were used for seven consecutive days. In addition, self-reports are informative about subjective intensity, while the cut-point values for accelerometer-measured MVPA do not usually take into account the fitness level of the user and may underestimate the amount of, at least, moderate intensity activity, especially among older adults [15, 17]. Moreover, in the samples used in this study, accelerometers are likely to have underestimated physical activity among persons who participated in cycling, water-based activities or upper-body training. Therefore, further studies on the role of personality in the discrepancy between physical activity measures are needed.

This study was limited by the absence of the other three Five Factor Model personality traits, namely conscientiousness, openness and agreeableness [9]. Because personality was not the primary focus in the ERMA and PASSWORD studies, longer questionnaires assessing all five personality traits were not included in the baseline questionnaires in either study. Previous studies have shown that both conscientiousness and openness are positively associated with physical activity [13, 14] and it would also have been interesting to study these traits. Additionally, the Five-Factor Model personality traits comprise lower-order facets, which offer more detailed information about the traits [46]. For example, the positive association between extraversion and physical activity is mainly explained by the facet of extraversion termed “activity” [20]. The inclusion of facet-level personality characteristics could have offered more information on the associations between personality and physical activity. Another weakness of this study was the relatively low internal consistency of the 19-item short form of the Eysenck Personality Inventory, especially in the ERMA sample; however, removing any item would not have improved the Cronbach’s alpha values. It is also possible that personality traits influence the willingness to participate in different types of trials. Unfortunately, information on those who declined to participate in the ERMA and PASSWORD studies was not available, and hence they cannot be compared on personality traits with the participants.

It is important to bear in mind that although identical measures of personality and self-reported physical activity were used in both samples, the accelerometers and ways of analysing raw accelerometer data differed between the two studies. Hence, the samples are not directly comparable in their absolute levels of accelerometer-measured physical activity. However, the accelerometer data were analysed with previously validated methods in both studies [35, 38] and it is unlikely that the slightly different cut-points for physical activity intensity would have affected the associations between personality traits and physical activity.

The use of two samples and of both accelerometers and self-reports to assess physical activity are the major strengths of this study. Although the PASSWORD sample was relatively inactive and the ERMA sample did not include men, both samples are large compared to previous studies using accelerometers [18–20, 22] and the results thus contribute to knowledge on the relationship between personality and different measures of physical activity. The ERMA sample showed relatively high amount of MVPA per day (whole day 49.7 ± 25.9 min) compared, for example, to Canadian women aged 40–59 (approximately 24 min/day) [3] and Norwegian women aged 20–64 (36 min/day) [4]. However, this seems to be in line with country-level comparisons showing that, among the high-income countries, Finland has one of the most physically active general populations [1]. The amount of MVPA in the ERMA sample was close to the mean of middle-aged women in Finland [47, 48] (see also Additional File 1).The results can be generalized to healthy middle-aged women and to sedentary or at most moderately active older adults.

## Conclusions

Individual differences in feeling, thinking, and behaving, captured by personality traits, help us to understand why some people are active, and some are not. In the representative sample of healthy Finnish middle-aged women, the negative association of neuroticism was seen in both self-reported physical activity and accelerometer-measured moderate-to-vigorous leisure time physical activity. In addition, among older adults high neuroticism was associated with underreporting physical activity compared to the accelerometers. More research is needed to understand the relationship between neuroticism and physical activity. However, the associations found among middle-aged women were not seen in the smaller sample of sedentary or at most moderately active older men and women. The correlates of physical activity in special groups like these warrant further research.

## Supplementary information

**Additional file 1: **Representativeness of the samples. Additional information about the representativeness of the samples. Includes **Table S1.** Comparison of education, marital status and body mass index (BMI) between the ERMA and PASSWORD study samples and the Finnish population; and **Table S2.** Comparison of study variables between participants who did and did not provide accelerometer or body mass index (BMI) data (mean ± standard deviation or frequency (%)).

**Additional file 2: Table S3.** Associations of extraversion and neuroticism with physical activity among middle-aged women (ERMA sample, *n* = 795–1093) and older men and women (PASSWORD sample, *n* = 293–314). Extraversion and neuroticism were tested in the same regression models. **Table S4.** Joint associations of extraversion and neuroticism with physical activity among middle-aged women and older adults. **Table S5.** Associations of extraversion and neuroticism with physical activity among middle-aged women and older adults. **Table S6.** Interaction effect of trait and sex on physical activity variables among older adults.

## Data Availability

Pseudonymized datasets are available on reasonable request. To request the data please contact Dr. Eija Laakkonen (eija.k.laakkonen@jyu.fi) (the ERMA data) or Prof. Sarianna Sipilä (Sarianna.sipila@jyu.fi) (the PASSWORD data).

## Abbreviations

BMI: Body mass index
CI: Confidence interval
ERMA: Estrogenic Regulation of Muscle Apoptosis
MET: Metabolic equivalent
MVPA: Moderate to vigorous physical activity
OR: Odds ratio
PASSWORD: Promoting safe walking among older people: the effects of a physical and cognitive training intervention vs. physical training alone on mobility and falls among older community-dwelling men and women
SD: Standard deviation
Yrs.: Years
