# Supplementary material for: Accelerometer-measured and self-reported physical activity in relation to extraversion and neuroticism: a cross-sectional analysis of two studies
Source: BMC Geriatr. 2020 Jul 29;20:264. doi: 10.1186/s12877-020-01669-7 (PMC7391808; doi:10.1186/s12877-020-01669-7)
Supplement: Supplementary file 1 — Additional file 1: Representativeness of the samples. Additional information about the representativeness of the samples. Includes Table S1. Comparison of education, marital status and body mass index (BMI) between the ERMA and PASSWORD study samples and the Finnish population; and Table S2. Comparison of study variables between participants who did and did not provide accelerometer or body mass index (BMI) data (mean ± standard deviation or frequency (%)). [file 12877_2020_1669_MOESM1_ESM.docx]

**Additional File 1**

**Representativeness of the samples**

The recruitment of the ERMA sample and the PASSWORD sample is briefly described in the manuscript and with more details in the protocol papers [1,2].

The comparison between the study samples and Finnish population in education, marital status and body mass index are shown in Table 1. Comparison between the samples and Finnish population in education were analyzed by Chi-Square tests and adjusted residuals. Statistical comparison in marital status was not reasonable because different categorization was used in the study samples and Finnish population sample. In addition, only information about mean body mass index (BMI) in Finnish population was available, hence statistical comparisons were not possible.

The ERMA baseline sample consisted of women aged 47 to 55 and can be considered to be representative of the healthy middle-aged female population in Finland [1]. Women who responded to the pre-questionnaire but did not continue to the baseline measurements had more often been oophorectomized and were less likely to use hormonal contraception than those who participated in the baseline measures [1]. This is not likely to effect the generalization of the results. The ERMA sample seems to be quite representative of the Finnish female population of the same age in marital status, but women with lower education were under-represented and women with higher education over-represented in the ERMA sample compared to the population sample. Additionally, the average body mass index seems to be slightly lower in the ERMA sample than in the Finnish population sample. This is likely to be due to the exclusion of severely obese participants (BMI > 35) from the study. Compared to the latest report of physical activity among Finnish population [3], the average amount of whole day moderate-to-vigorous physical activity was relatively similar in the ERMA sample (49.7 ±25.9 min/day) vs. 40-59 year-old female population (approximately 45 min/day).

The target group in the PASSWORD study was community-dwelling sedentary or at most moderately physically active men and women aged 70 to 85. The comparison between the study sample and Finnish population in education, marital status and body mass index is shown in Table 1. Both men and women with only comprehensive education were under-represented and with upper secondary education over-represented in the PASSWORD sample compared to the Finnish population sample. It seems that divorced men were under-represented and single women over- represented in the PASSWORD sample, but in general the PASSWORD sample was quite representative of the Finnish population in relation to marital status. Finnish population studies using accelerometer-based physical activity are restricted to younger population [3], but compared to the sample of 60-69 year-old Finnish adults (approximately 40 min/day moderate-to-vigorous physical activity), the PASSWORD sample is less physically active (32.5 ±20.1 min/day). Hence, the PASSWORD sample can be considered representative of Finnish sedentary or at most moderately active older men and women.

**Missing values and the differences between the analyzed sample and the recruited sample**

In the ERMA sample, acceptable accelerometer data were not available from 302 participants and information about BMI was missing from 171 participants. The participants who did not provide acceptable accelerometer data or BMI data were younger and scored higher in neuroticism compared to those from who acceptable accelerometer data or BMI data were available (Table 2). Walking speed (used in the sensitivity analysis) was missing from 116 participants. Women with missing walking speed were younger (50.1 ±2.0 vs. 51.0 ±2.1) and had higher BMI (26.1 ±4.0 vs. 25.4 ±3.7) than women with available walking speed data. All other variables had at most 1.5% of missing values.

In the PASSWORD data, acceptable accelerometer data were available from 293 participants. Women were less likely to provide acceptable accelerometer data than men, but otherwise there were no differences between the groups (Table 2). There was one missing value in the self-reported MET-minutes but otherwise information about all other study variables were available from all participants (n=314).

**References**

1. Kovanen V, Aukee P, Kokko K, Finni T, Tarkka IM, Tammelin T, et al. Design and protocol of Estrogenic Regulation of Muscle Apoptosis (ERMA) study with 47 to 55-year-old women’s cohort: novel results show menopause-related differences in blood count. Menopause N Y N. 2018;25:1020–32.

2. Sipilä S, Tirkkonen A, Hänninen T, Laukkanen P, Alen M, Fielding RA, et al. Promoting safe walking among older people: the effects of a physical and cognitive training intervention vs. physical training alone on mobility and falls among older community-dwelling men and women (the PASSWORD study): design and methods of a randomized controlled trial. BMC Geriatr. 2018;18:215.

3. Husu P, Tokola K, Suni J, Vähä-Ypyä H, Mänttäri A, Vasankari T, et al. Suomalaisten objektiivisesti mitattu fyysinen aktiivisuus, paikallaanolo ja fyysinen kunto. Opetus- ja kulttuuriministeriön julkaisuja 2018:30. Helsinki: Ministry of Education and Culture, Finland; 2018.

Table S1. Comparison of education, marital status and body mass index (BMI) between the ERMA and PASSWORD study samples and the Finnish population.

|  | ERMA women (n=1098) | Finnish women aged 45-54^1^ | PASSWORD women (n=188) | Finnish women aged 70+^1^ | PASSWORD men (n=126) | Finnish men aged 70+^1^ |
| --- | --- | --- | --- | --- | --- | --- |
| **Education %** |  |  |  |  |  |  |
| 1. comprehensive school | 2.2^a^ | 9.6 | 21.8^a^ | 51.8 | 24.6^a^ | 46.8 |
| 1. upper secondary school | 21.7^a^ | 38.0 | 54.2^b^ | 27.7 | 58.8^b^ | 26.5 |
| 1. post-secondary vocational college diploma | 34.7^b^ | 21.7 | 23.9^4^ | 20.5^4^ | 16.7^a,4^ | 29.8^4^ |
| 1. bachelor’s degree | 12.9 | 12.2 |  |  |  |  |
| 1. master’s degree | 23.6^b^ | 16.8 |  |  |  |  |
| 1. licentiate or doctoral degree | 4.9^b^ | 1.7 |  |  |  |  |
| **Marital status %** | ERMA women (n=1098) | Finnish women aged 47-55^2^ | PASSWORD women (n=188) | Finnish women aged 70-85^2^ | PASSWORD men (n=126) | Finnish men aged 70-85^2^ |
| Married | 61.5 | 55.5 | 47.9 | 44.5 | 77.8 | 67.1 |
| Single | 8.5 | 22.7 | 8.0 | 0.1 | 3.2 | 0.9 |
| Divorced | 14.6 | 20.1 | 17.6 | 17.6 | 4.8 | 14.0 |
| Widow | 0.8 | 1.6 | 20.2 | 29.4 | 8.7 | 9.5 |
| Cohabitant | 15.4 | - | 3.2 | - | 4.0 | - |
|  | ERMA women (n=926) | Finnish women aged  50-59^3^ | PASSWORD women n=188 | Finnish women aged 70-79^3^ | PASSWORD men n=126 | Finnish men aged 70-79^3^ |
| **BMI** mean | 25.5 | 28.1 | 28.0 | 28.3 | 27.9 | 27.4 |

^1^Resource: Official Statistics of Finland (OSF): Educational structure of population [e-publication]. ISSN=2242-2919. Helsinki: Statistics Finland [referred: 7.1.2020]. Access method: <http://www.stat.fi/til/vkour/index_en.html>.

^2^Resource: Official Statistics of Finland (OSF): Changes in marital status [e-publication]. ISSN=1797-643X. Helsinki: Statistics Finland [referred: 7.1.2020]. Access method: <http://www.stat.fi/til/ssaaty/index_en.html>.

^3^Resource: Koponen P, Borodulin K, Lundqvist A, Sääksjärvi K, Koskinen S. Terveys, toimintakyky ja hyvinvointi Suomessa – FinTerveys 2017-tutkimus. [Health, functional capacity and welfare in Finland – FinHealth 2017 study]. Report 4/2018. Helsinki: Terveyden ja hyvinvoinnin laitos; 2018.

^4^At least post-secondary vocational college diploma; education categories 3-6 combined.

^a^Under-presented and ^b^over-presented based on Chi-Square test and standardized residuals.

Table S2. Comparison of study variables between participants who did and did not provide accelerometer or body mass index (BMI) data (mean± standard deviation or frequency (%))

|  | ERMA study  (n=1098) | | | | | | PASSWORD study (n=314) | |
| --- | --- | --- | --- | --- | --- | --- | --- | --- |
|  | Accelerometer data | | | BMI data | | | Accelerometer data | |
|  | Yes (n=796) | No  (n=302) | Yes (n=927) | | No (n=171) | Yes  (n=293) | | No  (n=21) |
| Sex (female), n (%) | 765 (100) | 302 (100) | 927 (100) | | 171 (100) | 171 (58.4) | | 17 (81.0)^a^ |
| Age, yrs | 51.2 ±2.0 | 50.0 ±2.1^a^ | 51.0 ±2.0 | | 50.2 ±2.1^a^ | 74.0 ±3.8 | | 73.9 ±4.1 |
| BMI | 25.5 ±3.7 | 25.0 ±3.5 |  | |  | 27.9 ±4.8 | | 28.6 ±3.9 |
| Education (higher),  n (%) | 318 (39.9) | 137 (45.4) | 385 (41.5) | | 70 (40.9) | 64 (21.8) | | 2 (9.5) |
| Extraversion | 5.4 ±2.7 | 5.5 ±2.6 | 5.4 ±2.7 | | 5.6 ±2.7 | 4.5 ±2.7 | | 4.0 ±2.7 |
| Neuroticism | 2.8 ±2.1 | 3.1 ±2.2^a^ | 2.8 ±2.1 | | 3.3 ±2.3 | 3.1 ±2.3 | | 3.9 ±2.3 |
| Self-reported physical activity categories |  |  |  | |  |  | |  |
| Low, n (%) | 86 (10.8) | 34 (11.3) | 99 (10.7) | | 21 (12.3) | 115 (39.2) | | 11 (52.4) |
| Medium, n (%) | 213 (26.8) | 84 (27.8) | 262 (28.3) | | 35 (20.5) | 140 (47.8) | | 8 (38.1) |
| High, n (%) | 497 (62.4) | 184 (60.9) | 566 (61.1) | | 115 (67.3) | 38 (13.0) | | 2 (9.5) |
| Self-reported physical activity, MET-min/day | 216.5 ±214.2 | 243.4  ±243.3 | 221.3 ±220.0 | | 237.5 ±237.6 | 79.0 ±107.3 | | 85.7  ±118.4 |

^a^Statistically significant (p<.05) difference between the groups analyzed by independent samples t-test or Pearson chi-square test.
