# Supplementary material for: Accelerometer-measured and self-reported physical activity in relation to extraversion and neuroticism: a cross-sectional analysis of two studies
Source: BMC Geriatr. 2020 Jul 29;20:264. doi: 10.1186/s12877-020-01669-7 (PMC7391808; doi:10.1186/s12877-020-01669-7)
Supplement: Supplementary file 2 — Additional file 2: Table S3. Associations of extraversion and neuroticism with physical activity among middle-aged women (ERMA sample, n = 795–1093) and older men and women (PASSWORD sample, n = 293–314). Extraversion and neuroticism were tested in the same regression models. Table S4. Joint associations of extraversion and neuroticism with physical activity among middle-aged women and older adults. Table S5. Associations of extraversion and neuroticism with physical activity among middle-aged women and older adults. Table S6. Interaction effect of trait and sex on physical activity variables among older adults. [file 12877_2020_1669_MOESM2_ESM.docx]

Table S3. Associations of extraversion and neuroticism with physical activity among middle-aged women (ERMA sample, n=795–1093) and older men and women (PASSWORD sample, n=293–314). Extraversion and neuroticism were tested in the same regression models.

|  | Accelerometer | | | | | | | | Self-reported | | | |
| --- | --- | --- | --- | --- | --- | --- | --- | --- | --- | --- | --- | --- |
|  | Leisure time | | | | Whole day | | | | Leisure time | | | |
|  | Light PA | | MVPA | | Light PA | | MVPA | | MET | | PA level^a^ | |
|  | β | p | β | p | β | p | β | p | β | p | OR | 95% CI |
| ERMA study |  |  |  |  |  |  |  |  |  |  |  |  |
| Extraversion | .01 | .658 | .04 | .288 | .03 | .471 | .06 | .135 | .06 | .135 | 1.04 | .99-1.10 |
| Neuroticism | .02 | .381 | -.06 | .122 | .04 | .255 | -.03 | .470 | -.08 | .042 | .94 | .88-1.00 |
| PASSWORD study | | |  |  |  |  |  |  |  |  |  |  |
| Extraversion |  |  |  |  | .08 | .141 | .02 | .679 | -.06 | .320 | .95 | .87 – 1.04 |
| Neuroticism |  |  |  |  | .04 | .516 | .10 | .101 | -.06 | .339 | .97 | .87 – 1.07 |

Standardized Beta-coefficients (β) and p-values presented for linear regression analyses and odds ratios (OR) with 95% confidence interval (CI) for ordinal regression models. PA=physical activity, MVPA=Moderate-to-vigorous physical activity. Models adjusted for age, BMI and education. Models for accelerometer-based outcomes also adjusted for accelerometer wear time and model for light PA adjusted for MVPA and model for MVPA adjusted for light PA. Models for PASSWORD data also adjusted for sex. ^a^PA categories were low, medium and high (reference group).

Table S4. Joint associations of extraversion and neuroticism with physical activity among middle-aged women and older adults.

|  | Accelerometer | | | | | | | | Self-reported | | |
| --- | --- | --- | --- | --- | --- | --- | --- | --- | --- | --- | --- |
|  | Leisure time | | | | Whole day | | | | Leisure time | | |
|  | Light PA | | MVPA | | Light PA | | MVPA | | MET | | PA level |
|  | Partial  η2 | p | Partial  η2 | p | Partial  η2 | p | Partial  η2 | p | Partial  η2 | p | p |
| ERMA study | |  |  |  |  |  |  |  |  |  |  |
| E&N | .001 | .678 | .007 | .062 | .002 | .484 | .006 | .122 | .008 | .045 | .010 |
| PASSWORD study | | |  |  |  |  |  |  |  |  |  |
| E&N |  |  |  |  | .007 | .336 | .008 | .251 | .004 | .501 | .489 |

E=extraversion, N=neuroticism. Wald test for significant impact from predictor combination.

Table S5. Associations of extraversion and neuroticism with physical activity among middle-aged women and older adults.

|  | Accelerometer-based physical activity | | | | | | | | Self-reported physical activity | | | |
| --- | --- | --- | --- | --- | --- | --- | --- | --- | --- | --- | --- | --- |
|  | Leisure time | | | | Whole day | | | | Leisure time | | | |
|  | Light PA | | MVPA | | Light PA | | MVPA | | MET | | PA categories^a^ | |
|  | β | p | β | p | Β | p | β | p | β | p | OR | 95% CI |
| ERMA study | |  |  |  |  |  |  |  |  |  |  |  |
| Extraversion | .00 | .931 | .06 | .112 | .01 | .763 | .06 | .101 | .09 | .008 | 1.05 | 1.00–1.11 |
| Neuroticism | .02 | .443 | -.06 | .081 | .03 | .346 | -.04 | .301 | -.07 | .053 | 0.94 | 0.88– 1.01 |
| PASSWORD study | | |  |  |  |  |  |  |  |  |  |  |
| Extraversion |  |  |  |  | -.04 | .640 | -.03 | .666 | -.05 | .401 | .95 | .87–1.03 |
| Neuroticism |  |  |  |  | .02 | .804 | .09 | .074 | -.02 | .731 | 1.01 | .92–1.11 |

Extraversion and neuroticism were tested in the separate regression models. Standardized Beta-coefficients (β) and p-values are presented for linear regression analyses and odds ratios (OR) with 95% confidence interval (CI) for ordinal regression models. PA=physical activity, MVPA=Moderate-to-vigorous physical activity. Models adjusted for age, BMI and education. Models for accelerometer-based outcomes also adjusted for accelerometer wear time and model for light PA adjusted for MVPA and model for MVPA adjusted for light PA. Models for PASSWORD data also adjusted for sex, chronic diseases and walking speed and models for ERMA data adjusted for menopausal status, chronic diseases and walking speed. Model for ERMA leisure time also adjusted for employment status. ^a^PA categories were low, medium and high (reference group).

Table S6. Interaction effect of trait and sex on physical activity variables among older adults.

|  | Accelerometer-based physical activity | | | | Self-reported physical activity | | | |
| --- | --- | --- | --- | --- | --- | --- | --- | --- |
|  | Light PA | | MVPA | | MET | | PA categories^a^ | |
|  | Β | p | β | p | β | p | OR | 95% CI |
| Extraversion | -.02 | .834 | -.02 | .802 | -.03 | .687 | .96 | .87 – 1.07 |
| Sex | .11 | .241 | -.07 | .508 | .01 | .911 | 1.05 | .45 – 2.45 |
| Extraversion * Sex | .16 | .151 | .02 | .898 | -.01 | .953 | .99 | .84 – 1.16 |
| Neuroticism | .02 | .751 | -.03 | .747 | -.06 | .465 | 1.07 | .95 – 1.22 |
| Sex | .25 | .003 | -.20 | .031 | -.02 | .800 | 1.73 | .83 – 3.61 |
| Neuroticism * Sex | -.04 | .730 | .21 | .061 | .05 | .673 | .84 | .70 – 1.02 |

Extraversion and neuroticism were tested in the separate regression models. Standardized Beta-coefficients (β) and p-values are presented for linear regression analyses and odds ratios (OR) with 95% confidence interval (CI) for ordinal regression models. PA=physical activity, MVPA=Moderate-to-vigorous physical activity. Models adjusted for age, BMI and education. Models for accelerometer-based outcomes also adjusted for accelerometer wear time and model for light PA adjusted for MVPA and model for MVPA adjusted for light PA. ^a^PA categories were low, medium and high (reference group).
